# Electroacupuncture Enhances Cognitive Deficits in a Rat Model of Rapid Eye Movement Sleep Deprivation via Targeting MiR-132

**DOI:** 10.1155/2022/7044208

**Published:** 2022-09-16

**Authors:** Li Hao, Yiming Wu, Jin Xie, Xinwang Chen

**Affiliations:** ^1^Medical College, Henan University of Chinese Medicine, Zhengzhou 450046, Henan, China; ^2^College of Acupuncture-Moxibustion and Tuina, Henan University of Chinese Medicine, Zhengzhou 450004, Henan, China

## Abstract

Deprivation of rapid eye movement sleep (REMSD) reduces the potential for learning and memory. The neuronal foundation of cognitive performance is synapse plasticity. MicroRNA-132 (MiR-132) is an important microRNA related to cognitive and synapse plasticity. Acupuncture is effective at improving cognitive impairment caused by sleep deprivation. Furthermore, its underlying principle is still unclear. Herein, whether electroacupuncture (EA) helps alleviate cognitive impairment in REMSD by targeting miR-132 was assessed. A rat model of REMSD was constructed using the developing multiplatform water environment technique, as well as EA therapy in Baihui (GV20) and Dazhui (GV14) was performed for 15 minutes, once daily for 7 days. Agomir or antagomir of MiR-132 was injected into the hippocampal CA1 areas to assess the EA mechanism in rats with REMSD. Then, the learning and memory abilities were detected by behavioral tests; synapse structure was assessed by transmission electron microscope (TCM); and dendrites branches and length were examined by Golgi staining. MiR-132-3p was assessed by real-time quantitative polymerase chain reaction (qRT-PCR). P250GAP, ras-related C3 botulinum toxin substrate 1 (Rac1), and cell division cycle 42 (Cdc42) expression levels in hippocampal tissues were evaluated by immunohistochemistry and Western blot. According to the research, EA therapy enhanced cognitive in REMSD rats, as evidenced by reduced escape latency; upregulated the performance of platform crossings and prolonged duration in the goal region; and improved spontaneous alternation. EA administration restored synaptic and dendritic structural damage in hippocampal neurons, enhanced miR-132 expression, and reduced p250GAP mRNA and protein levels. Additionally, EA boosted the protein level of Rac1 and Cdc42 associated with synaptic plasticity. MiR-132 agomir enhanced this effect, whereas miR-13 antagomir reversed this action. The current data demonstrate that EA at GV20 and GV14 attenuates cognitive impairment and modulates synaptic plasticity in hippocampal neurons via miR-132 in a sleep-deprived rat model.

## 1. Introduction

Sleep is indeed a fundamental necessity for sustaining a healthy brain [1]. Rapid eye movement (REM) sleep makes up the majority of mammalian sleep. REM is a physiological process necessary for brain development, spatial memory acquisition, consolidation, and recovery of physical functions [2–4]. It has been demonstrated that REM sleep deprivation (REMSD) contributes to cognitive impairment [5, 6]. Synaptic plasticity is considered to be neurological for cognition. The hippocampus becomes an essential part of the nervous system related to learning and memory [7–9]. The impact of REMSD on learning and memory often reflects changes in the synaptic plasticity of hippocampal neurons [10–14]. Additionally, long-term SD increases the risk of insomnia and dementia. Consequently, it seems to be of considerable clinical value to investigate how SD impairs learning and memory and find effective treatment methods.

MicroRNAs (miRNAs or miRs) comprise small segments of short non-coding RNA chains averaging 21–22 nucleotides in length relevant to the occurrence, differentiation, migration, and fusion of neurons. They either inhibit mRNA transcription or directly guide the degradation of target mRNA [15]. MicroRNA-132 (MiR-132), an endogenous microRNA, participates in the control of neural differentiation or maturation, axonal growth, neural migration, and neuronal plasticity. It targets and regulates several downstream molecules, affecting neurons' physiological and pathological functions. MiR-132 can influence the pathogenesis of several diseases of the nervous system, such as Alzheimer's disease, epilepsy, and sleep deprivation [16–19]. Karabulut et al. demonstrated that REMSD caused downregulation of miR-132 and impairs spatial cognitive [6]. The survey indicated that miR-132 performs a major function in the growth as well as the progression of dendritic spines [20]. Knockdown of miR-132 shortens the dendritic length and reduces branching, causing reduced synaptic plasticity and decreased learning and memory capacity [21]. Davis et al. noted that intracerebroventricular injections of miRNA-132 mimic prolonged the duration of rapid eye movement sleep (REMS) [22]. These hint that sleep deprivation altered miRNA-132 levels in the brain and could affect learning and memory capacities.

As a traditional Chinese medical treatment, acupuncture is one of the most widely accepted alternative medicine both domestically and internationally. In recent years, acupuncture has been used in insomnia clinically [23–25]. Cumulative findings suggest that acupuncture decreases cortical excitability and may improve sleep deprivation-induced cognitive deficits by regulating proteins linked to hippocampus synaptic function [26, 27]. However, the molecular mechanism of miR-132 in acupuncture to improve learning and memory in REMSD is still not explained. Accordingly, the current study aimed to investigate whether EA therapy alleviates spatial cognitive deficits and synaptic plasticity by targeting miR-132 in the hippocampus of the REMSD animal model.

## 2. Materials and Methods

### 2.1. Animals

Male Sprague Dawley rats, 2 months old (220–250 g), were acquired from Henan University of Chinese Medicine (licence number: SYXK (HA) 2020-0004). All animals were maintained under regulated conditions, a 12 h:12 h light:dark schedule; the temperature ranged between 21°C and 23°C, with a humidity level of 50% to 60%; and food and water were available at any time. Related research procedures involving animals in the present investigation were confirmed by the Institutional Animal Care Committee of Henan University of Chinese Medicine (approval number: DWLL20210309) and conform to the guidelines of the National Institute of Health (NIH) guideline.

Six groups of 12 rats each were established from a random distribution of 72 rats. (1) the animals divided into the control condition were treated using an improving multiplatform water environment method without electroacupuncture (EA); (2) the model group's animals underwent through improved multiplatform water environment method; (3) the EA group's animals got EA treatment; (4) non-EA animals were provided EA administration across non-acupoints; (5) the rats divided into the miR-132-agomir group received EA treatment after miR-132-agomir microinjection; and (6) the rats in the miR-132-antagomir group received EA treatment after miR-132-antagomir microinjection.

### 2.2. Stereotaxic Injection

miR-132-agomir and miR-132-antagomir (Guangzhou RiboBio Co. Ltd., Guangzhou, China) were dissolved in 0.01 M PBS to the concentration of 1 nmol/*μ*L and 0.5 nmol/*μ*L, respectively. Following anesthesia, the stereotactic device was used to position the animals, and the symmetrical hippocampal CA1 areas (4.0 mm lateral from bregma, 2 mm toward the parietal centerline, as well as 2.5 mm depth) were selected as suitable sites for miR-132-agomir and miR-132-antagomir microinjections with 5 *μ*l on each side. The injections were performed within 15 min and left to stand for 5 min. Animals including the control, models, EA, and non-EA treatments were administered with equal sterile saline into the relative hippocampus.

### 2.3. Sleep Deprivation Paradigm

Sleep deprivation was commenced 7 days after recovery from the stereotaxic injection. This experiment used an improved multiplatform water environment method to induce REM sleep deprivation [28]. The rats in the model, EA, non-EA, miR-132-agomir, and miR-132-antagomir groups were housed 20 hours per day (9:00 a.m. the previous day to 5:00 p.m. the next day) [29] for 7 days inside a container that is 110 cm in length, 60 cm in width, as well as a 40 cm relatively high. Ten small cylindrical platforms, including a 3 cm radius as well as an 8 cm height were positioned inside the water chamber, with 15 cm separating each platform from the next. Within the chamber, rats were placed on a small platform 1 cm beyond the level of the water. Whenever rats on the platform entered REM sleep, a decrease in muscle tone caused their faces to make contact with water and then rouse, thus achieving sleep deprivation. The animals divided into the control condition were housed on a sizable platform with a 9 cm radius, where the rats could move freely and sleep normally. All rats were acclimatized for 2 hours per day for 3 days before the experiment, which was designed to keep the rats balanced on the platform.

### 2.4. Electroacupuncture Treatment

The acupoints used in the present research were derived from the Standard Acupuncture Nomenclature. The locations of acupoints used in this study correspond to human anatomical regions [30]. EA therapy was carried out at 5:30 p.m. each day for 15 minutes, once daily for 7 consecutive days. Sterile and disposable acupuncture needles were attached to the outlet of the EA equipment (G6805; Suzhou Medical Products Factory Co. Ltd., China), and 2 Hz continuous wave and 1 mA current were employed.

The rats in the EA, miR-132-agomir, and miR-132-antagomir groups were given acupuncture needles (Suzhou Medical, China) of 0.25 mm diameter and 25 mm long at acupoints. The depth of acupuncture was 5 mm. The non-EA animals were administered using needles at 5 mm next to GV20 and GV14, that is, at non-acupoints, attached to the electroacupuncture apparatus rather unenergized.

### 2.5. Morris Water Maze (MWM)

**Figure 1:**
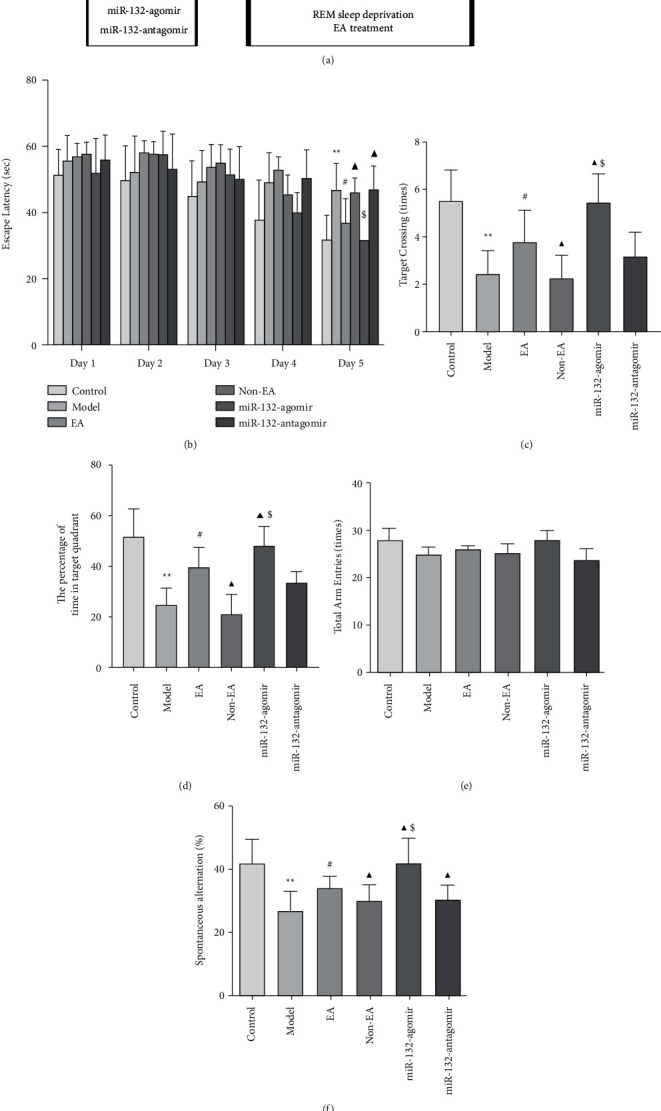
EA improved spatial learning and memory in REMSD rats (a), schematic diagram of the experimental protocol (b), the escape latency spent in the MWM test was used to assess acquisition in the training trials (c, d), the number of platform crossings and percentage of time spent in the target quadrant of rats in each group were used to detect memory in probe tests in MWM test (e, f), the total arm entries and spontaneous alternation of rats in each group were used to assess working memory in the Y maze. Data were presented as the mean ± s.d, Two-way ANOVA was used for panels (b), and One-way ANOVA was used for panels (c–f), respectively. ^∗^*P* < 0.05 versus control group; ^#^*P* < 0.05 versus model group; ^▲^P < 0.05 versus EA group; $P < 0.05 vs. Non-EA group. n =12 per group.

MWM (Roto-Rod, Ruiwode, China) was utilized to measurehippocampal-dependent spatial learning and memory of REMSD rats from day 9 to day 14 in this study (Figure 1(a)). The experimental equipment included a cylindrical water tank (100 cm in diameter and 50 cm in height) and an escape platform (10 cm in diameter). The reservoir of water, which was separated into four quadrants, was filled with water that was invisible to the milk powder. The circular platform was concealed at 1 cm beneath the water level. The MWM test comprised two parts: the training and the probe tests.

Training trials were performed from day 9 to day 13, during which the platform was also concealed inside the identical region. Each rat faced that tank wall, falling into the liquid, yet was permitted to explore that concealed destination in 60 seconds. As that platform was not found, the rat was led to it and remained on it for a brief period of time. The escape duration was observed and analyzed.

Probe tests were performed on day 14 of EA treatment. The platform was removed, and the number of platform crossings and percentage of time spent in the target quadrant were measured and analyzed. A total of 12 rats per group were used in this test.

TEM results illustrated that EA had a beneficial effect on the synaptic ultrastructure of hippocampal neurons in the REMSD model rats, which was correlated with the activation of miR-132.

### 2.6. Y Labyrinth Test

Y labyrinth (Roto-Rod, Ruiwode Life Technology Co. Ltd.) was conducted to assess the overall impact of EA on the ability of cognitive in animals, The Y labyrinth consists of three identical support arms and a middle triangle connection, with which the arm angle is 120°. The three arms are marked as Nos. 1, 2, and 3. The rat was placed at the middle triangle junction facing the No. 1 arm during the experiment, and the software was tracked and recorded. The rat moved freely in a non-interference environment, and the recording time was 5 minutes. At the end of the experiment, each rat was wiped in time and sprayed with 75% ethanol to eliminate odor interference. When the rat enters three different arms continuously, it is one accurate alternation. Spontaneous alternation = (accurate alternation times/total arm insertion times – 2) × 100%. After the experiment, the total number of arm advances and spontaneous alternation were examined by employing the Smart software (Panlab SL, Barcelona, Spain).

### 2.7. Sample Preparation

All animals were sacrificed after behavior tests. After anesthetization with 20% (w/v) uratan, the whole brains of six rats were eliminated instantly, and the hippocampus was excised and preserved for qRT-PCR, Western blot, and Golgi staining individually. Three rats per group were transcardially perfused with saline and 4% glutaraldehyde. The hippocampus was removed, cut into 1 mm^3^ tissue pieces, and post-fixed for 4 hours at 4°C for the transmission electron microscope. For immunohistochemistry, the last three rats per group were transcardially perfused with saline and paraformaldehyde.

### 2.8. Transmission Electron Microscope

TCM examined the synaptic connections between neurons. Tissue pieces were embedded, post-fixed, and dehydrated in the order listed. Then the samples were soaked in acetone at 37°C for 24 hours. Ultrathin sections of 70 nm had been generated and positioned using copper grids. Sections were examined using TCM (1400, Japan Electronics, Japan) at 100 kV. The Henan University of Chinese Medicine performed TCM services for Advanced Microscopy. Three rats per group were used in this test.

### 2.9. Quantitative Real-Time Polymerase Chain Reaction (qRT-PCR)

This technique was utilized to examine miR-132-3p and p250GAP mRNA expression. RNA production from hippocampal tissue of the brain was performed with TRIzol (15596026, Invitrogen, USA). cDNA was generated utilizing a suitable reagent (RR037, Takara, Dalian, China). Related equipment (ABI7500, Applied Biosystems, USA) was used to detect miR-132-3p and p250GAP mRNA using a kit (RR420, Takara, China). The amplification process appeared like this: 10 minutes at 95°C to denaturate, subsequently 40 cycles, including 15 seconds of denaturation at 95°C, 30 seconds of annealing at 60°C, as well as 1 minute of extension at 60°C. GAPDH as well as U6 were used as internal controls. The primers (Sangon Biotech, China) are as follow: miR-132-3p (forward: 5′-ACACTCCAGCTGGGTAACAGTCTACAGCCA-3), p250GAP (forward:5′-ATGGAGATCGACAATAAGGGAAAC-3′, reverse: 5′- GACGCAATGACCAGGGAAGAG-3′), GAPDH (forward: 5′- CTGGAGAAACCTGCCAAGTATG-3′, reverse: 5′-GGTGGAAGAATGGGAGTTGCT-3′), and U6 (forward: 5′-CTCGCTTCGGCAGCACA-3′, reverse: 5′-AACGCTTCACGAATTTGCGT-3′). The p250GAP mRNA or miR-132-3p amounts were calculated using ^ΔΔ^Ct (threshold cycle, Ct) values normalized to GAPDH and U6.

### 2.10. Immunohistochemistry

This method was employed to determine the level of ras-related C3 botulinum toxin substrate 1 (Rac1) as well as cell division cycle 42 (Cdc42) in the hippocampus. The paraffin-embedded tissue was divided into 5 *μ*m coronal sections by a slicer (VT 1200s, Leica, Germany) before being treated in goat serum (SL038, Solarbio, China). The sections were exposed to the primary antibodies p250GAP (1:50, bs-9296R, Bioss, China), Racl (1:50, ab33186, Abcam, UK), and Cdc42 (1:20, ab155940, Abcam, UK). Then, the sections were washed using PBS, and the secondary antibody was treated for 1 hour at around 22°C. The tissues were given 3,3′-diaminobenzidine tetrahydrochloride (DAB) for color development and detected (Axioscope 5, ZEISS, Germany). Image software was used to measure the result of hippocampal cells.

### 2.11. Western Blot

This method was utilized to measure the expression of Racl and Cdc42. Frozen hippocampal tissue was lysed with detergent (R0010, Solarbio, Beijing, China) containing an inhibitor of proteases phenyl-methylsulfonyl fluoride (P0100, Solarbio, Beijing, China) and phosphatase inhibitors. The supernatant was collected after centrifugation at 12,000 rpm for 10 minutes at 4°C, and the protein concentration was measured according to the BCA assay kit instructions (PC0020, Solarbio, Beijing, China). Equivalent volumes of SDS-PAGE sample buffer (P1200, Solarbio, Beijing, China) were mixed into the protein supernatant and denatured completely by boiling at 100°C for 5 minutes. The buffer was loaded onto 10% SDS-polyacrylamide gels in different lanes for electrophoresis. Following electrophoresis, the proteins were transferred by electroblotting onto polyvinylidene difluoride (PVDF; IPVH00010, 0.45 *μ*m, Merck Millipore, MA, USA). The membranes were closed with 5% skimmed milk in TBST (TBS plus 0.05% Tween-20) for 1 hour. They were then incubated overnight at 4°C with primary antibodies Racl (1:1,000, ab33186, Abcam, Cambridge, UK), Cdc42 (1:1,000, ab155940, Abcam, Cambridge, UK), and *β*-actin (1:2,000, ab8226, Abcam, Cambridge, UK). The membrane was washed three times with TBST and then incubated for 2 hours with secondary antibody goat anti-rabbit IgG (1:5,000, ab6721, Abcam, Cambridge, UK). According to the manufacturer's instructions, an appropriate amount of ECL-enhanced chemiluminescence (P0018AS, Beyotime, Shanghai, China) was mixed and dropped onto the front of the film. All bands were photographed and visualized by Image J v1.51 (National Institutes of Health, Bethesda, MD, USA).

### 2.12. Data Analyses

Research findings were all presented according to mean ± standard deviation. The discrepancies were investigated using a two-tailed unpaired Student's *t*-test or a parametric one-way analysis of variance (ANOVA). Behavioral evaluations from day 8 to day 12 were measured using two-way ANOVA. The threshold for significance was always fixed at *P* < 0.05. Statistical treatment was performed using Prism 8.0.1 (GraphPad Software, USA).

## 3. Results

### 3.1. EA Improved Cognitive Abilities in REMSD Rats

MWM was performed for assessing the effects of EA Baihui (GV20) and Dazhui (GV14) on the spatial cognitive of REMSD rats. In comparison to the control, the rats in the model group had prolonged escape latency (*P* < 0.01) on day 5 of the training exam and significantly reduced performance of platform crossings and prolonged duration in the goal region in the probe exam (*P* < 0.01). In comparison to the model, EA shortened the escape latency and increased the performance of platform crossings and prolonged duration in the goal region (*P* < 0.05); non-EA did not differ from the model (*P* > 0.05). Nevertheless, miR-132-agomir, miR-132-antagomir, and non-EA improved and reversed these metrics, respectively, in comparison to EA (*P* < 0.05). In addition, miR-132-agomir decreased and elevated these indices, and miR-132-antagomir had a non-significant effect in contrast with the non-EA group (*P* < 0.05, *P* > 0.05; Figures 1(b)–1(d)).

The Y maze was utilized for measuring the impact of EA on the working memory of REMSD rats. Research data showed no variation in the total arm entries among the groups (*P* > 0.05; Figure 1(e)). However, in comparison to control, reduced spontaneous alternation was observed in the model animals (*P* < 0.01). EA treatment enhanced the spontaneous alternation (*P* < 0.05), while non-EA had no action (*P* > 0.05). It is essential to know that in comparison to EA, miR-132-agomir and miR-132-antagomir significantly enhanced and decreased the spontaneous alternation separately. No noticeable variation was identified involving the model as well as the EA groupings (*P* > 0.05). Besides this, miR-132-agomir raised the spontaneous alternation (*P* < 0.05), and miR-132-antagomir had no meaningful effect (*P* > 0.05), in comparison to the non-EA group (Figure 1(f)).

Behavioral data indicates that EA can ameliorate REMSD-induced cognitive deficits and is perhaps connected with miR-132.

### 3.2. EA Alleviated Synaptic Ultrastructure Damage in REMSD Model Rats

Research has shown that learning and memory impairment in sleeping deprivation were complemented by modifications to the synaptic structure [31]. Thus, this study evaluated the synapse morphological ultrastructure of the rats' hippocampal neurons through TEM. The data demonstrated that the presynaptic membrane (PM) and post-synaptic membrane (PD) were thinned and blurred, the synaptic gap was narrowed, and the synaptic interface was shortened in the model in comparison to the control rats. While EA attenuated damage to synaptic ultrastructure, PM and PD thickened and cleared; the synaptic gap became wider; and synaptic interface length increased. The model and the non-EA rat models displayed no distinctions (*P* > 0.05).

**Figure 2:**
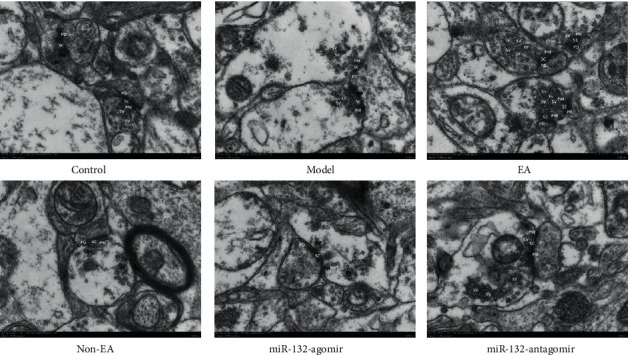
EA ameliorated damage to neuronal synaptic structures in REMSD rats. Transmission electron microscope detection of synapse in the CA1 regions of the hippocampus in each group. Scale bars, 500 nm. Presynaptic membrane (PM), post-synaptic membrane (PD) and synaptic vesicle (SV) showed in the representative pictures. n = 3 per group.

Furthermore, we showed that the ameliorative actions of the EA were improved through miR-132-agomir, while miR-132-antagomir and non-EA attenuated the actions of the EA. Compared with the non-EA group, miR-132-agomir significantly mitigated lesions in synaptic ultrastructure, while miR-132-antagomir had no apparent role (Figure 2).

TEM and Golgi staining findings suggested that EA treatment improved the structural synaptic plasticity of hippocampal neurons in sleep-deprived rats related to miR-132.

### 3.3. EA Protected Dendrite in REMSD Model Rats

**Figure 3:**
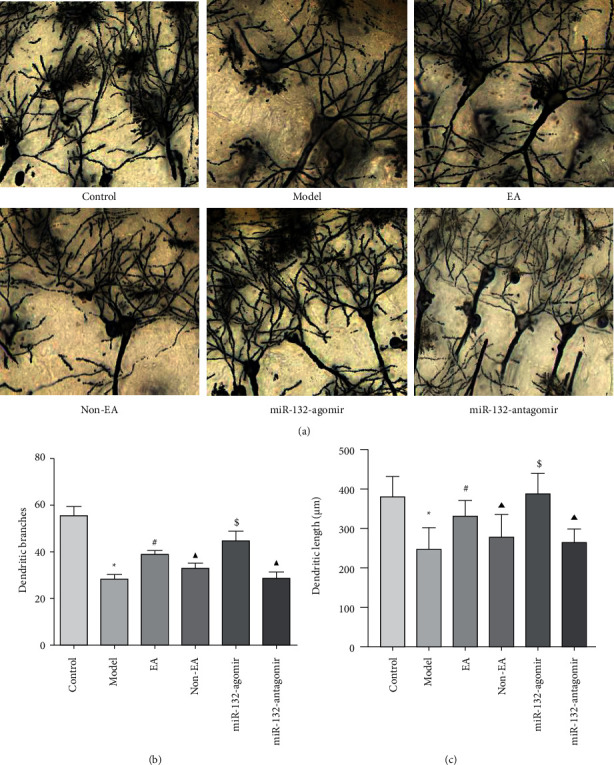
EA attenuated lesions of neuronal dendritic structures in the hippocampus of REMSD Model Rats (a), representative pictures of Golgi staining in each group, scale bar, 100 *μ*m (b, c), dendritic branches and dendritic length in each group were measured by Image J software. Data were presented as the mean ± s.d. One-way ANOVA was used for panels (a–c). ^∗^*P* < 0.05 versus control group; ^#^*P* < 0.05 versus model group; ^▲^*P* < 0.05 versus EA group; and $ *P* < 0.05 versus non-EA group. n = 3 per group.

Dendrites are an important site of synaptic plasticity. Dendritic branches and length in the hippocampus are essential for spatial cognition, which are highly sensitive to stress [32, 33]. To recognize more about how EA influences dendritic branch and length, we detected the morphology of CA1 neurons in the hippocampus by Golgi staining. In comparison to control rats, dendritic branches and length declined in model animals (*P* < 0.05). EA administration upregulated the dendritic branches and length (*P* < 0.05). As opposed to EA administration, miR-132-antagomir as well as non-EA downregulated dendrite branching and length (*P* < 0.05), whereas miR-132-agomir tended to upregulate it, without statistical comparison (*P* > 0.05). When compared to the model animal, the non-EA rat exhibited no discrepancy (*P* > 0.05). Additionally, in comparison to non-EA administration, miR-132-agomir showed an increment in dendritic branches and length (*P* < 0.05), while miR-132-antagomir performed a non-significant effect (*P* > 0.05; Figures 3(a)–3(c)).

### 3.4. Impact of EA on the miR-132-3p and p250GAP in REMSD Model Rats

**Figure 4:**
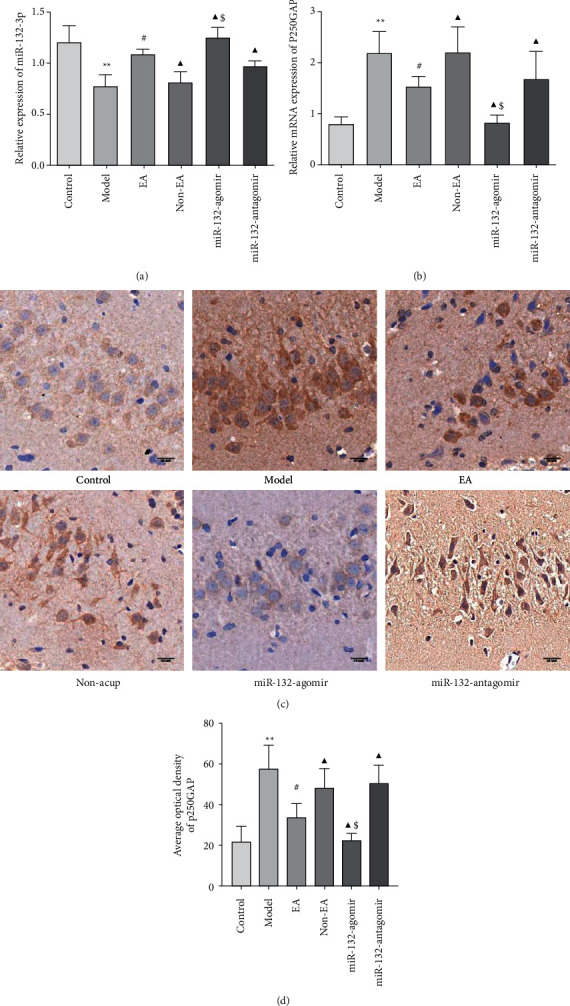
Effect of EA on the miR-132-3p and p250GAP in REMSD model rats (a), the miR-132-3p expression level in each group and (b), quantification of p250GAP mRNA expression in each group by qRT-PCR (c), the representative pictures of p250GAP immunohistochemistry staining. Scale bars, 50 *μ*m (d), the average optical density of p250GAP was measured by Image J software in the hippocampal slices. Data were presented as the mean ± s.d. One-way ANOVA was used for panels (a–d). ^∗^*P* < 0.05, *P* < 0.01 versus control group; ^#^*P* < 0.05 versus model group; ^▲^*P* < 0.05 versus EA group; $*P* < 0.05 vs. Non-EA group. n = 3 per group.

Mammalian miR-132 is formed by two homologous miRNAs on the same chromosome. MiR-132-3p has developed into a critical component of the miR-132/212 family [34]. MiR-132-3p had a vital function in the modulation of neuronal synaptic proteins in hippocampal neurons [35]. To better understand the basic principles behind EA enhancing structural synaptic plasticity, we stereotactically injected miR-132-3p agomir and antagomir into the hippocampus individually. Then, qRT-PCR was used to evaluate miR-132-3p. Our outcome revealed that miR-132-3p expression decreased in model rats in comparison to the normal rat (*P* < 0.05). However, EA enhanced the levels of miR-132-3p (*P* < 0.05). When compared to EA rats, the miR-132-3p agomir, miR-132-3p antagomir, and non-EA increased and decreased miR-132-3p levels in the hippocampus (*P* < 0.05). Nevertheless, non-EA and model groups did not differ from one another (*P* > 0.05). Besides, miR-132-agomir significantly heightened miR-132-3p levels in comparison to the non-EA rat (*P* < 0.05), while miR-132-antagomir had no appreciable role (*P* > 0.05; Figure 4(a)).

Evidence has shown that miR-132 and its target gene p250GAP are involved in dendrite growth and synapse formation [36, 37]. Accordingly, we investigated p250GAP levels using qRT-PCR and immunohistochemistry, separately. The immunohistochemistry data revealed that p250GAP positive expression was mainly in the cytoplasm around the nucleus, which was dark yellow or brown, and the nucleus was blue (Figure 4(c)). The outcome of qRT-PCR and immunohistochemistry indicated that EA treatment decreased p250GAP mRNA and the average optical density when compared to the model rat (*P* < 0.05). Compared to the EA treatment, miR-132-3p agomir, miR-132-3p antagomir, and non-EA declined and promoted p250GAP mRNA and protein expression separately (*P* < 0.05). Non-EA and model groups did not differ from one another (*P* > 0.05). Furthermore, miR-132-agomir markedly declined p250GAP mRNA and mean optical density in comparison to the non-EA (*P* < 0.05), while the role of miR-132-antagomir was no variation (*P* > 0.05; Figures 4(b)–4(d)).

Above-described data suggested that p250GAP mRNA and its protein were generally consistent. EA alleviated structural synaptic plasticity by modulating miR-132-3p and p250GAP in REMSD model rats.

### 3.5. EA Enhanced the Expression of Rac1and Cdc42 Protein in REMSD Model Rats

**Figure 5:**
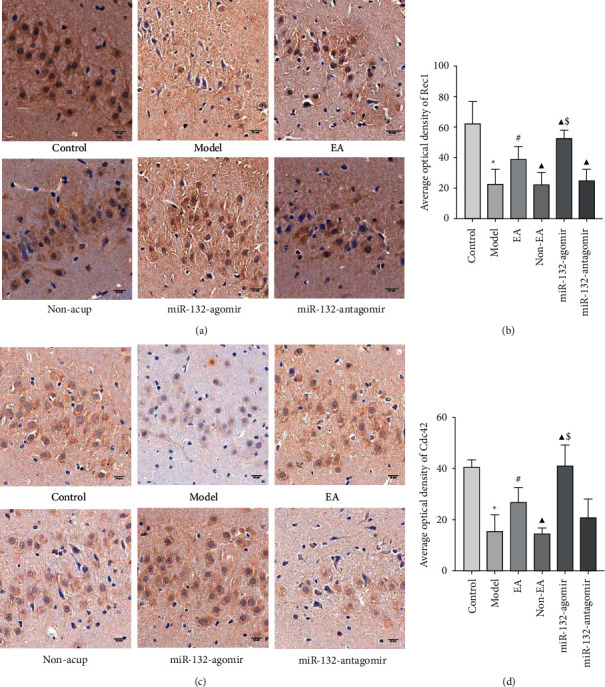
EA increased the protein expression of Rac1and Cdc42 in the hippocampus of REMSD rats (a, c), representative pictures of Immunohistochemistry staining in the hippocampal slices of each group of rats, scale bars, 50 μm (b, d), the average optical density of Rac1and Cdc42 in each group were measured by Image J software. Data were presented as the mean ± s.d. One-way ANOVA was used for panels (a–c). ^∗^*P* < 0.05 versus control group; ^#^*P* < 0.05 versus model group; ^▲^*P* < 0.05 versus EA group; and $ *P* < 0.05 versus non-EA group. n = 3 per group.

**Figure 6:**
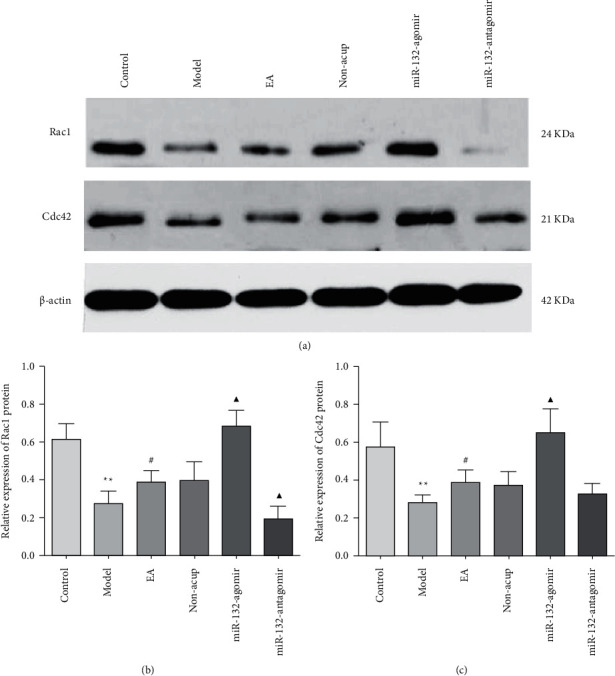
EA enhanced the protein expression of Rac1and Cdc42 in the hippocampus of REMSD rats (a–c), protein levels of Rac1 and Cdc42 in each group were detected by western blot, and *β*-actin was used as a loading control. Data were presented as the mean ± s.d, One-way ANOVA was used for panels (a–c). ^∗^*P* < 0.01 versus control group; ^#^*P* < 0.05 versus model group; ^▲^*P* < 0.05 versus EA group; $*P* <0.05 vs. Non-EA group. n = 3 per group.

p250GAP mainly impacts the formation of dendritic spines as well as synapses by targeting the inhibition of Rac1 and Cdc42. Rac1 is the primary initiating factor for synapse formation. Cdc42 also performs an essential function in regulating synaptic maturation as well as plasticity changes. Did EA therapy affect the expression levels of Cdc42 and Rac1? To shed light on this issue, we investigated the expression of Rac1 and Cdc42. The data indicated that in comparison to the control, the expression of Rac1 and Cdc42 was reduced in hippocampal tissues of rats in the model animal (*P* < 0.05). EA treatment elevated the levels of Rac1 and Cdc42 (*P* < 0.05). Compared to EA therapy, miR-132-3p agomir, miR-132-3p antagomir, and non-EA up- and downregulated the expression of Rac1 and Cdc42, respectively (*P* < 0.05). Additionally, miR-132-agomir upregulated Rac1 and Cdc42 levels remarkably compared to non-EA administration (*P* < 0.05), whereas miR-132-antagomir had no effect (*P* > 0.05; Figures 5(a)–5(d) and 6(a)–6(c)). Such findings revealed that EA increased Rac1 and Cdc42 expression and linked it to miR-132.

## 4. Discussion

EA has proven to improve the decline in learning and memory caused by sleep deprivation [38]; however, the explanation remains unidentified. Our present research determined that electroacupuncture alleviated learning and memory deficits and improved hippocampal neurons' synaptic plasticity in the REMSD rat model. Furthermore, this role was related to the targeted regulation of miR-132.

In this study, the non-EA group was added in order to objectively evaluate the effectiveness of EA. We initially detected the effect of EA on cognition in REMSD animals through the Morris water maze and Y maze. Related results revealed that electroacupuncture could shorten the escape latency of REMSD rats, extend the stay time in the target quadrant, and increase the number of crossing platforms. The alternation rate of REMSD rats was increased by EA stimulation in the Y maze. However, non-EA reverted these indices. These results indicated that EA alleviated REMSD-induced learning and memory deficits.

Synapses are unique connections between neurons that are used to transmit information and nerve impulses. To explore the molecular mechanism of EA treatment to ameliorate REMSD-related learning and memory impairment, we examined the ultrastructure of neuronal synapses in the CA1 area by TEM. In some REMSD rats, the structure of synapses PM and PD in the hippocampal CA1 area blurred; the membrane became thinner; the synaptic gap disappeared or narrowed; and the synaptic interface shortened. The number of synaptic vesicles was significantly reduced. EA and miR-132 agomir can make the structure of PM and PD clear; the synaptic interface was extended; and the number of SVs increases, whereas non-EA treatment reversed these metrics. The outcome indicated EA's improvement in cognitive may be relevant to ameliorating synaptic ultrastructure impairment.

Synaptic plasticity refers to changes in synaptic structure and function. Due to the fact that dendrites are smaller, more numerous, and more sensitive to stimuli than axons, they are an important component of synaptic plasticity. To study EA's impact on synaptic plasticity, we followed by observing dendrites of neurons in the CA1 region of the hippocampus by Golgi staining. The results demonstrated that EA can upregulate the number of dendritic branches and length of neurons in the hippocampal CA1 area of sleep-deprived rats, and miR-132 agomir could reinforce this effect. There was a significant difference in the non-EA effect compared with the EA, which means that non-EA can downregulate the number of dendritic branches and the length of neurons in sleep-deprived rats. Therefore, EA modulates structural synaptic plasticity in neurons associated with sleep deprivation by activating miR-132.

MiR-132 has a correlation with synapse formation. The report found that miR-132 elevated in the hippocampus of rats after learning. High miR-132 production in hippocampus neurons will increase dendritic spines and strengthen synaptic transmission [39]. Knockout of miR-132 in vivo can decrease synapses, the release of synaptic neurotransmitters, and memory formation. Therefore, we hypothesized that EA improves learning and memory impairments caused by sleep deprivation concerning miR-132. To test this hypothesis, miR132-agomir and miR132-antagomir were injected into the hippocampus to make miR-132 overexpression and underexpression. Behavioral results showed that overexpression of miR-132 in hippocampal neurons enhanced EA treatment on improving cognitive caused by sleep deprivation, and knockdown of miR-132 reduced the effect of EA on learning and memory. Besides, overexpression of miR-132 enhanced the therapeutic efficacy of EA, as evidenced by a clear structure and increased dendritic branching and length of hippocampal neuronal synapses.

Studies have revealed that miR-132 mainly regulates the formation of dendritic spines and synaptic plasticity through the regulation of its downstream target genes. The currently evident miR-132 target genes include p250GAP, MeCP2, and MMP-9 [40]. The upregulation of miR-132 can inhibit p250GAP protein; that is, miR-132 is a negative regulator of p250GAP [37]. P250GAP is a RhoGAP protein mainly expressed in the cerebrum and is one of the target proteins downstream of miR-132 [41]. Overexpression of p250GAP can reduce dendritic branching and length and dendritic spine density, leading to synaptic plasticity changes [42]. MiR-132 is an essential factor that regulates synaptic plasticity. Its increase mediates p250GAP activity inhibition, promotes the formation of dendritic spines in the hippocampus, affects the morphological changes of synaptic structure, and improves cognitive function.

Rac1 is a component in the Rho GTPase family, which increases the dendritic branches and length and dendritic spine density, and it is the primary initiating factor for synapse formation [43]. Haditsch et al. [44] observed that mouse Rac1 mutations led to learning and memory decline. Martinez and Tejada-Simon [45] believed that inhibiting Rac1 can damage LTP's induction, and activating Rac1 can enhance synaptic transmission excitability and synaptic plasticity. Cdc42 regulates the cytoskeletal protein actin and axon growth [46]. The mutation and decreased expression of Cdc42 may affect the development of the dendritic spine and synaptic plasticity, causing the decline of learning and memory [47, 48]. To further explore the mechanism of miR-132 in improving sleep deprivation-induced cognition by EA, we tested the expression of p250GAP, Rac1, and Cdc42. We observed that EA reduced the p250GAP level and increased Rac1 and Cdc42. The improvement effect of EA is more apparent when miR-132 is overexpressed, which shows that overexpression of miR-132 can inhibit p250GAP and enhance the levels of Rac1 and Cdc42. Meanwhile, the outcome of Western blot displayed that the difference of Rac1 and Cdc42 in rat hippocampus between EA and non-EA groups showed no statistical significance, hinting that our subsequent study could be explored from the perspective of mRNA.

In summary, our research demonstrates EA therapy has an ameliorative action on SD-induced cognitive deficits and synaptic plasticity, which is associated with targeted activation of miR-132. These results demonstrate that EA represents a prospective neuroprotective therapy with promising clinical applications for neurological diseases such as sleep deprivation.

## Data Availability

The data utilized and evaluated in this investigation are provided upon reasonable request from the corresponding author.
